# Beyond the diagnosis: gender disparities in the social and emotional impact of cancer

**DOI:** 10.1590/1806-9282.2024S115

**Published:** 2024-06-07

**Authors:** Mariana Seabra Leite Praça, Frederico Timm Rodrigues de Sousa, Eduardo Batista Cândido, Rívia Mara Lamaita, Maria Celeste Osório Wender, Agnaldo Lopes Silva

**Affiliations:** 1 Universidade Federal de Minas Gerais, Department of Obstetrics and Gynecology – Belo Horizonte (MG), Brazil.; 2 Universidade Federal de Minas Gerais, Clinical Hospital – Belo Horizonte (MG), Brazil.; 3 Universidade Federal do Rio Grande do Sul, Department of Gynecology and Obstetrics – Porto Alegre (RS), Brazil.

## INTRODUCTION

A diagnosis of cancer not only triggers physical challenges and profound emotional distress but also creates an increased demand for healthcare resources. The surge in psychological symptoms, particularly anxiety and depression, not only worsens morbidity but also correlates with suboptimal clinical outcomes and diminished quality of life for survivors. This emotional burden and the subsequent need for social adjustment often diverge along gender lines, revealing a complex interplay between gender-specific social roles and stress and adjustment processes^1^.

Navigating the unexplored landscapes of their new reality, cancer patients derive substantial benefits from questioning traditional societal expectations, particularly in the realm of caregiving. This essential shift calls for a more extensive embrace, notably from male partners, as it confronts ingrained gender norms. Fostering a more inclusive approach to caregiving not only fortifies emotional support during trying times but also possesses the potential to instigate transformative changes in social identity, contesting deeply rooted gender roles^2^.

As the global rise in cancer survivor numbers continues, driven by an aging population, enhanced early detection, and groundbreaking treatments, the aftereffects of cancer therapies cast long shadows over survivors' lives. Understanding gender disparities and their social repercussions, including financial consequences impacting health-related quality of life, becomes crucial for developing strategies attuned to gender nuances^3,4^.

Cancer ranks among the leading causes of premature mortality in women globally, featuring prominently in the top three in nearly all countries. Despite this, the global focus on women's health often centers around reproductive and maternal aspects, reflecting a patriarchal framework that aligns with narrow, anti-feminist views of women's worth and societal roles^5^.

The aftermath of cancer therapies extends far beyond the cessation of treatments, leaving a lasting impact on the lives of survivors. These lingering effects cast long shadows over their well-being, underscoring the importance of recognizing and addressing the distinct challenges faced by individuals of different genders. This recognition becomes a cornerstone for developing more targeted and supportive interventions that cater to the diverse needs of cancer patients and survivors^6^.

This article delves into the intricacies of gender differences in the social impact of cancer, exploring psychological adjustment, sexual intimacy challenges, marital dynamics, and the enduring disparities in healthcare access. Additionally, it highlights the complexities of life beyond cancer treatment, emphasizing the need for a holistic approach to care that considers emotional, behavioral, and social factors. The implications for clinical practice and avenues for future research underscore the importance of continuous efforts to improve healthcare equity and outcomes for all individuals affected by cancer.

## UNRAVELING GENDER DIFFERENCES IN THE SOCIAL IMPACT OF CANCER

Gender is not merely a biological distinction; it encompasses a broad spectrum of socially constructed roles, relationships, and individual characteristics such as personality traits, attitudes, behaviors, and values. These aspects, which are differentially applied to and internalized by men and women, are deeply entrenched within our societal fabric^7^. Such constructs invariably shape the psychological and social responses to health challenges, particularly in the context of a life-altering illness like cancer.

The literature has consistently illuminated the influence of gender roles and societal expectations on the health behaviors of men and women, especially in the coping mechanisms employed when faced with chronic diseases^8^. It is commonly posited that women, whose societal roles often resonate with acceptance and nurturing, may navigate the vicissitudes of chronic illness with a certain grace, perhaps due to a closer alignment with the conventional feminine archetype^9^.

Typically, women are perceived to adopt emotional coping strategies, expressing, and processing their emotions more openly. Conversely, men are often inclined toward a problem-solving approach, seeking solutions, and occasionally resorting to avoidance as a coping mechanism. The expected patient role, necessitating emotional expression and reliance on others, poses a greater challenge for men, who are socially conditioned to value rationality and independence. Traditionally seen as more relational and interdependent, women might find a semblance of comfort in the socio-emotional patient role.

Notably, these gender-driven differences in psychological adjustment seem to converge when patients face the rigors of treatments such as chemotherapy or confront advanced disease stages. At this juncture, both genders report a marked increase in psychological distress, a testament to the leveling nature of severe health adversity^9^.

The ripple effects of a cancer diagnosis extend beyond the individual to the family unit, impacting the relational dynamics and well-being of caregivers and other family members. Cancer's reach spares none, challenging the resilience and adaptive capacities of all involved^10,11^.

In the intimate dance of managing cancer within a partnership, gender emerges as a significant determinant of distress levels, transcending the roles of patient or caregiver. Strikingly, women report higher levels of distress than men, a difference that is consistent across various research methodologies and unaffected by variables such as time since diagnosis or sample demographics^12^.

Considering these insights, supportive care must be envisioned as an all-encompassing strategy. It should provide support to individuals with cancer and their families throughout the entire cancer journey, from the shock of the initial diagnosis to the uncertainties of treatment, the ups and downs of recovery, the trials of chronic management, and the finality of end-of-life care, extending into the bereavement process^13^.

## INFLUENCE OF RACE AND BIOLOGICAL FACTORS.

The etiology of disparities in cancer treatment and outcomes can be attributed to a complex interplay of factors, including race, ethnicity, cultural differences, socioeconomic status, and educational background. Geographic variations in healthcare provider and hospital standards, as well as biological distinctions among different ethnic groups, must also be considered. Additionally, non-adherence to evidence-based treatment guidelines has been identified as a modifiable factor that can lead to worse survival outcomes^14^.

Due to variations in survival outcomes among minority patients, there has been a growing emphasis on recruiting them for clinical trials. This focus aims to uncover inherent differences in tumor biology, treatment responses, and survival, particularly in controlled treatment regimens between different patient groups. There is a lack of minority enrollment in gynecologic cancer clinical trials. A paper examining 170 Gynecologic Oncology Group (GOG) trials conducted between 1994 and 2013 reported that out of the 45,259 patients involved in these trials, 83% were White, 8% were Black, and 9% were classified as "other" (p<0.01)^15^. The Center for Disease Control (CDC) age-adjusted incidence data found that the enrollment of Black patients was significantly lower than expected for various types of gynecologic cancer trials. For example, it was 15 times lower than expected for ovarian cancer trials, 10 times lower for endometrial cancer trials, 4.5 times lower for cervical cancer trials, and 5.2 times lower for sarcoma trials (p<0.001), regardless of the type of study or the publication year^14^.

The natural history of cancer has several critical junctures: prevention [human papillomavirus (HPV) vaccination], risk factor acquisition (obesity), screening (cervical cytology), diagnosis (endometrial biopsy for postmenopausal bleeding), and treatment (appropriate surgery by appropriate provider). Racial variation at any point along this course might contribute to minor disparate outcomes, while even subtle inequities at multiple nodes might accumulate to create more glaring gaps in outcomes^16^.

The cause of existing disparities is multifaceted, and so will be the solution. As the World Health Organization clearly states, cervical cancer can be eradicated through addressing several main pillars: ensuring equal access and ready availability of the HPV vaccine to vulnerable populations, stressing the importance of cervical cancer screening, facilitating follow-up of abnormal cytology results, and employing guideline-concurrent therapies in the treatment of cervical cancer. Similarly, adherence whenever possible to evidence-based guidelines would likely diminish disparities in survival among ovarian cancer patients^16^.

While endometrial cancer presents unique challenges, there are clear opportunities for improved practice. Physicians must be vigilant about counseling patients on the importance of reporting postmenopausal bleeding to their physicians. Physicians should in turn have a high level of suspicion when evaluating Black women, particularly of an older age, with abnormal uterine bleeding, knowing current evaluation algorithms may offer significantly lower sensitivity for cancer in this population^16^.

## COMPLEXITIES OF SEXUAL INTIMACY AFTER CANCER

Cancer's assault on the body extends beyond the physiological, delving deeply into the personal realm of sexual intimacy. The metamorphosis in physical appearance and function, which is a result of aggressive cancer treatments, carries significant emotional and psychological weight. Body image dissatisfaction and sexual dysfunction rank among the most profound challenges faced by those on the cancer journey^17^.

The overall prevalence of sexual dysfunction among female cancer survivors ranged from 16.7 to 67%. Patients and their intimate partners often find themselves in a maelstrom of sexuality-related issues, which is a struggle that persists through the illness and often into the recovery phase. Regrettably, a substantial number of patients are left unprepared for the sexual alterations that may arise and lack the essential information and support to navigate these changes effectively.

Sexual issues are frequently overlooked and inadequately addressed in routine healthcare practice, representing an unmet need that significantly impacts the overall health and quality of life of cancer survivors. Proactive engagement by healthcare professionals in couple's communication and strategic interventions is crucial. Such initiatives are foundational in fostering a mutual understanding of the sexual modifications following cancer, potentially safeguarding the quality of the relationship^18^.

Gynecologic cancer survivors are susceptible to a heightened risk of sexual dysfunction and body image concerns due to the direct impact of treatment on genital anatomy and hormonal milieu. This vulnerability can surface immediately post-treatment or linger into the long-term survivorship phase. Recent endeavors in the medical community have pivoted toward recognizing and ameliorating these quality-of-life concerns for these survivors^19^.

The prevalence and severity of psychosexual dysfunction following radical interventions, such as vulvectomy, are stark, with nearly half of the patients reporting a decline in the quality of their sexual relationships^20^. Pelvic radiotherapy, while lifesaving, is not without its drawbacks, often leading to complications such as vaginal stenosis, dryness, and vulvodynia, which can drive patients toward psychological avoidance of sexual contact to elude pain and discomfort^20^.

Surgical treatments, especially those involving the pelvis, can severely disrupt a patient's self-perception. Procedures that result in anatomical alterations, such as vaginal shortening and vulva deformities, can instigate fears surrounding pain during intercourse, impacting the patient's sexual health even in the absence of physical sequelae^21^.

The hormonal repercussions of oophorectomy, particularly when bilateral, are profound, inducing immediate surgical menopause with all its attendant symptoms—hot flashes, vaginal dryness, and sleep disturbances. These physical manifestations are mirrored by a decline in sexual satisfaction for a significant portion of those who undergo the procedure^22,23^.

Furthermore, the impact on fertility and the sense of femininity is acutely felt by younger women, instilling fears, and insecurities about resuming sexual activity. These anxieties, coupled with the emotional weight of fertility loss, can erode self-confidence and dampen sexual desire^20^.

Chemotherapy introduces its own set of challenges, with common side effects such as nausea and hair loss. For women, in particular, hair is a pillar of self-identity, and its loss can have a disproportionately negative impact on self-esteem and body image^21,24^.

## MARITAL TIES IN THE SHADOW OF CANCER: DIVORCE RATES AND RELATIONSHIP DYNAMICS

The crucible of cancer diagnosis exerts a profound influence on couples, reshaping the dynamics of their relationship in fundamental ways. Even when the prognosis is not dire, the ramifications of cancer diagnosis and subsequent treatment phases can be transformative for both the patient and their partner. They become mutual pillars of support, intertwining their coping mechanisms, and providing both emotional and practical support on this arduous journey^25^.

Marriage can be a fortress of social and emotional support for those battling cancer, offering vital resources during treatment and convalescence, such as financial stability through a spouse's income and health insurance benefits. Yet, the tremors of a cancer diagnosis can precipitate marital discord, as couples grapple with the evolving health landscape, emotional upheaval, and financial strain. The specter of long-term sequelae and chronic conditions stemming from cancer treatment, especially for younger survivors, can impinge on their general well-being, sexual health, and reproductive potential^26^.

Statistical analysis, adjusted for demographic factors, reveals that young cancer survivors bear a 77% heightened risk of divorce or separation compared with their healthy counterparts. This risk is particularly acute among young female survivors, who are 80% more likely to experience marital dissolution than their non-cancer-afflicted peers^27^. Conversely, among older adults, evidence suggests that cancer does not significantly impact the likelihood of divorce^26^.

In the daily rhythm of family life, responsibilities such as childcare and housework frequently fall disproportionately on women. A cancer diagnosis has the potential to intensify this imbalance, especially in younger families where the female partner is the one facing the illness. Such a situation strains the fabric of the family, disrupting the traditional caregiver role and adding complexity to the already demanding dynamics^28^.

Infertility can stem from the cancer itself, surgical procedures, or as a consequence of gonadotoxic treatments such as chemotherapy and radiotherapy^29^. Fertility concerns and sexual dysfunction can be pivotal in destabilizing marriages, especially among younger couples who are navigating the complexities of conception. These issues can exert additional emotional and psychological pressure on the relationship, underscoring the intricate nexus between health, intimacy, and marital stability in the shadow of cancer^30^. Whether fertility is permanently compromised or temporarily impaired, the associated concerns contribute to heightened levels of anxiety, depression, grief, and stress, ultimately decreasing the overall quality of life^29^.

There is emerging evidence suggesting that individuals, particularly those dealing with cancer-related fertility concerns, may harbor fears of abandonment or rejection by current or future partners. Additionally, they may experience a reduction in sexual satisfaction and encounter difficulties or even breakdowns in their relationships. This highlights the need for more focused research and support to address the unique challenges that cancer-related fertility concerns pose to the dynamics of couple relationships.

Approximately 21% of diagnosed cases of gynecological cancer involve women of childbearing age. As survival rates rise, preserving fertility in reproductive-age women becomes crucial for enhancing quality of life. Fertility-sparing surgical approaches and assisted reproductive technologies (ARTs), including ovarian transposition and cryopreservation of oocytes or embryos, are preferred strategies. Emerging techniques, such as antiapoptotic/cell-preserving agents and stem cell technologies, are advancing the field of fertility preservation^31^.

A binational study from Denmark and Sweden highlights an increased prevalence of mental health disorders among spouses of cancer patients, with a 30% surge in the risk of first-onset mental health conditions, predominantly within the initial year post-diagnosis. This increase is particularly notable for depressive and stress-related disorders, illustrating the substantial psychological burden borne by partners of cancer patients^32^.

## PSYCHOLOGICAL AND EMOTIONAL RESILIENCE

Approximately 9.2 million new cases of cancer and 4.4 million cancer-related deaths were estimated among women of all ages in 2020^5^. The psychological impact of a cancer diagnosis reverberates profoundly, spanning from the turbulent treatment phase into the uncertain aftermath, presenting unique challenges for women at various stages. Patients grapple with a spectrum of emotions, including stress, anxiety, depression, sexual dysfunction, and sleep deprivation^32^.

Fear of cancer recurrence emerges as a pervasive emotion, casting its shadow across all patients, irrespective of prognosis. Those with a severe fear report constant, intrusive thoughts about cancer, a steadfast belief in its inevitable return regardless of the current prognosis, and an inability to plan for the future. Elevated levels of this fear can significantly compromise quality of life, alter health service utilization, and prevent adherence to follow-up protocols.

In the face of these challenges, individual resilience becomes a beacon of hope. It means an individual's ability to adapt positively to adversity, trauma, and various pressures. Cultures worldwide have recognized resilience as a powerful personal resource to navigate stressful situations, offering potentially positive effects on quality of life and overall adjustment, particularly when coping with cancer.

Looking ahead, as the number of cancer survivors is projected to soar to 26.1 million by 2040, this resilient community confronts enduring challenges. The repercussions—physical, emotional, and financial—persist throughout survivorship. Notably, 60% contend with persistent distress and an unsettling fear of recurrence. Additionally, around 36.5% find themselves unable to return to work, while between 15 and 75% navigate the intricate terrain of cancer-related cognitive impairment. Gender assumes significance in the broader context of the psychological and social adjustment process for cancer patients^6^.

Understanding the nuanced impact of gender on the social adjustment and psychological distress experienced by cancer patients and their spouses enriches our understanding of the multifaceted challenges individuals and couples encounter in the aftermath of cancer^33^.

## DISPARITIES IN HEALTHCARE ACCESS

Inequality profoundly impacts access to healthcare across various societal levels, affecting both resource availability and information accessibility, especially for women facing cancers undergoing complex, multimodal therapy, and frequent interactions with the healthcare system. Vulnerable populations with basic social resource needs may not receive optimal care, even when recommended by their healthcare provider. Recognizing and incorporating these factors into the formulation of public policies is vital for achieving a more effective and cost-efficient healthcare approach.

Across the globe, a woman's geographical location (including the country, region, or local environment concerning the proximity of healthcare services) and her socioeconomic status significantly influence the likelihood of developing any cancers. Factors such as poverty and social disenfranchisement play a pivotal role in determining the timing of her presentation to healthcare services and the accessibility of affordable, high-quality diagnostic and treatment options. Addressing these disparities is crucial, and one powerful strategy lies in the widespread implementation of preventive measures, like HPV vaccination.

Unfortunately, many women face limited opportunities to benefit from these life-saving interventions. In numerous resource-poor regions within countries, the availability and accessibility of early detection programs, cancer surgery, essential cancer treatment, palliative care, and support for cancer survivors, often termed as survivorship care, are inadequate. These discrepancies contribute to a scenario where disability and premature death resulting from preventable diseases such as breast or cervical cancer become tragically inevitable^34^.

Furthermore, within the domain of women's health, disparities disproportionately impact sexual and racial minorities. A study in the United States revealed that women from sexual minority backgrounds faced a higher likelihood of experiencing adverse health outcomes, surpassing even their sexual minority male counterparts. Among the demographic groups scrutinized, Hispanic women from sexual minority backgrounds exhibited the highest prevalence of negative outcomes, with alarming rates reaching 59% for depression, 41% for poor mental health, and 41% for poor physical health^35^.

This inequality extends beyond health outcomes and permeates socio-cultural and political dimensions. Historical instances, such as the apartheid era, underscore the absence of public policies for cervical cancer screening, exacerbating disparities. During that period, screening efforts were confined to family planning clinics, conducted in an unorganized manner, and lacking a defined age group, thereby perpetuating a cycle of inequality in healthcare access. Addressing these multifaceted disparities is essential to ensuring equitable health outcomes for all women, regardless of their sexual orientation or racial background^36^.

Numerous studies have explored issues related to cancer information access. One study revealed that men reported higher satisfaction with the information they received, suggesting a potential disparity in the quality of information received by men and women. The importance of this observation lies in acknowledging information as a fundamental tool for enhancing healthcare quality. Successful information dissemination is correlated with improved diagnoses and greater efficacy of screening programs.

In numerous countries worldwide, irrespective of their geographic location or economic resources, women often find themselves at a disadvantage compared with men, lacking both the knowledge and the authority to make informed decisions regarding their healthcare. The choice of language in addressing this issue is of utmost importance. Adopting gender-transformative approaches can shift the narrative from assigning blame to women for "late presentation," "neglected cancer," or "treatment abandonment." Instead, it emphasizes the value of women, recognizing their diverse experiences, as equal counterparts to men. This approach acknowledges women as individuals with agency and knowledge, empowering them to make evidence-based, informed decisions about their own healthcare^5^.

Raising public awareness about breast cancer can lead to earlier diagnoses. Further supporting this, in Thailand, where 97% of the population has access to cervical cancer screening, individuals with higher education levels demonstrate two to four times greater engagement. This highlights the crucial role of information in enhancing the quality of healthcare^37^.

To institutionalize this transformative perspective, an intersectional gender-transformative competency framework can be integrated into the education and training of the global cancer workforce. This ensures that healthcare professionals are equipped with the skills necessary to provide high-quality and respectful care that acknowledges the diverse needs of all individuals. By fostering a culture of equality and empowerment, we can strive to create an environment where women are actively engaged in their healthcare decisions, contributing to improved overall health outcomes and a more equitable healthcare landscape globally^5^.

One of the first steps of any cancer control effort is to define the cancer burden. Regrettably, less than 20% of cancer patients reside in areas equipped with a cancer registry. In low- and middle-income countries, evidence indicates a deficiency in cancer registries, which compromises the development of public policies relying on biased and unrepresentative data. Compounding this challenge, patients in these regions face the additional burden of inadequate treatment, with an estimated 70% of breast cancer patients grappling with this issue^38^.

## SURVIVAL AND BEYOND

Recovery from cancer encompasses more than just physical healing; it is also shaped by emotional, behavioral, and social factors.

The stigmas do not cease after the end of treatment, even people with clinical cure criteria present sequelae from the disease and the treatments undergone. A Chinese systematic review identified the main unmet care needs, among them social support (74%), difficulty with daily activities (54%), worsening of sexual/intimate function (52%), fear of recurrence/spread (50%), and information support (45%)^39^. Furthermore, survivors deal with chronic pain, sleep problems, substance abuse, sexual issues, and loss of physical abilities/functions^40,41^.

One study indicated that one of the main unmet demands of oncology patients is emotional support, which highlights the importance of including psychological care in post-treatment follow-up. Moreover, many patients experience chronic physical sequelae even after being cured, such as lymphedema, chronic treatment-related pain, insomnia, and even metabolic syndrome. It is necessary to consider the particularities of post-cancer patients and value their complaints, always remembering that they are patients with a chronically weakened psychological state, and in large part, they have repressed demands that should be addressed in a welcoming and humanized environment^40,41^.

Considering that the economic cost of cancer accounts for up to 4% of the global gross domestic product (GDP), governments should view addressing the global burden of women's cancers as a wise investment. This investment is particularly crucial considering the significant impact of these cancers on premature death and disability, leading to enduring social, financial, and economic consequences for affected women, their immediate families, and broader communities^34^.

The macroeconomic consequences of women's cancers are noteworthy, extending their influence on the national economy and society. This impact manifests through heightened health expenditures, losses in labor and productivity, and diminished investments in human and physical capital formation^34^.

At the microeconomic level, cancer profoundly affects women, their families, individual firms, and governments. Notably, a substantial portion of women's work goes beyond monetary transactions and is thus unlikely to be reflected in conventional macroeconomic indicators. Any calculations seeking to evaluate the economic burden associated with women's cancer should encompass non-income-generating activities, such as gathering water and firewood, preparing food, tending to livestock, and caring for children^5,34^.

When it comes to social support, it is important to consider the financial aspect of the patient's life. A study pointed out that cancer survivors have a 37% higher relative risk of not returning to work activities compared with people without a cancer diagnosis.

Looking beyond the individual effects on women and incorporating their families, the probability of catastrophic expenditures is alarmingly high in low-resource settings. Frequently, families faced with substantial direct and indirect costs linked to cancer and its treatment find themselves compelled to sell assets and accumulate debts. This already challenging situation is often exacerbated by employment-related complications, including decreased productivity, job loss, dismissal, and a reduction in work-related benefits. Addressing the economic impact of women's cancers is not only a health imperative but also an economic necessity for sustainable development^5^.

It is necessary for healthcare professionals to be aware of the main challenges that oncology patients face even after treatment so that they can provide appropriate support. Furthermore, it is essential to understand that cancer treatment itself, including radiotherapy, chemotherapy, surgeries, hormonal therapies, and monoclonal antibodies, is only part of the process, and there should be a holistic view of the cancer patient, considering both the treatment and disease-related sequelae.

## IMPLICATIONS FOR CLINICAL PRACTICE

Considering the points discussed thus far, it is possible to propose practical recommendations for the care of women undergoing cancer treatment. Considering the informational aspect, it is essential to provide space for patients to ask questions and express their needs. Especially for patients from racial and social minorities, higher rates of mental and physical disorders persist even after cancer treatment. Therefore, demonstrating concern for their needs can have a positive impact on recognizing these situations, allowing them to be addressed in the therapeutic plan once identified.

Furthermore, in a globalized world, it is important to consider socio-cultural aspects when approaching patients. For example, in Asia, the peak incidence of breast cancer occurs at an earlier age, typically between 45 and 55 years, while in the Western world, the incidence tends to rise post-menopause. Additionally, Indigenous and African American patients, as well as individuals of African descent, have a higher incidence of aggressive tumors such as triple-negative breast cancer^36^.

Moreover, in this regard when it comes to nutrition, the Western traditional diet is rich in salt and fat, high glycemic index carbohydrates, and red meat as the most used protein, these aspects are highly correlated with increased rates of endometrial, breast, and colorectal cancer. It is estimated that dietary improvements can reduce the incidence of colorectal cancer by up to 70%.

## FUTURE DIRECTIONS FOR RESEARCH

The future of cancer patient care is intricately linked to continuous research, leading to increasingly effective treatments with more manageable side effects. Despite this progress, substantial research gaps persist. Notably, it is crucial to highlight that only 5% of cancer research investment is directed toward developing and underdeveloped countries, despite these nations bearing 65% of all cancer-related deaths^42^.

Gender inequality is apparent in the populations studied for oncology trials. Among the 5,157 patients in trials leading to FDA approval of 17 new drugs in 2018, only 38% were women^42^. In clinical trials conducted from 2003 to 2016 (excluding prostate and breast cancer trials), women were underrepresented in studies on lung cancer, melanoma, and pancreatic cancer, despite the significant prevalence of these cancers in the female population^43^.

Furthermore, when it comes to gynecological cancers, it is evident that minorities and elderly women have been underrepresented in studies. Between 1985 and 2012, elderly individuals accounted for only 17% of participants in breast cancer studies, even though 42% of all breast neoplasms diagnosed annually are in this population^36^.

It is essential to continue advocating for this issue because research outcomes are influenced by the population involved in the study. Studies need to accurately represent the real-world profile of the population that will receive the medications under investigation. This ensures that treatments are effective and safe for all individuals, regardless of gender, age, or minority status. Closing the research gap and achieving better representation in clinical trials are crucial steps toward improving healthcare equity and outcomes for all.

## CONCLUSION

The individual impacts of cancer on the female population are crucial to examine, as a comprehensive understanding of these intricacies can significantly enhance our approach to addressing the unique challenges faced by women. This article contributes to ongoing efforts by delving into the gender-specific ramifications of cancer on social impact, sexuality, divorce rates, and other dimensions. By doing so, it supports the goal of providing comprehensive and gender-sensitive care to individuals navigating the complexities of cancer diagnosis, treatment, and survival.

Looking ahead, it is imperative to work toward mitigating social inequality in access to treatments and the conduct of clinical trials. This proactive approach can ensure that study populations more accurately reflect the diversity of real populations, ultimately advancing the effectiveness and inclusivity of cancer research and care.
